# Developmental grey matter changes in superior parietal cortex accompany improved transitive reasoning

**DOI:** 10.1080/13546783.2018.1481144

**Published:** 2018-10-03

**Authors:** Cristián Modroño, Gorka Navarrete, Antoinette Nicolle, José Luis González-Mora, Kathleen W. Smith, Miriam Marling, Vinod Goel

**Affiliations:** a Departamento de Ciencias Médicas Básicas, Facultad de Ciencias de La Salud, Universidad de La Laguna (ULL), Campus de Ofra, San Cristóbal de La Laguna (Tenerife), España, Spain;; b Center for Social and Cognitive Neuroscience (CSCN), School of Psychology, Universidad Adolfo Ibáñez, Santiago de Chile, Chile;; c Department of Psychology, York University, Toronto, Ontario, Canada

**Keywords:** MRI, transitive reasoning, VBM, parietal cortex, development, logical reasoning

## Abstract

The neural basis of developmental changes in transitive reasoning in parietal regions was examined, using voxel-based morphometry. Young adolescents and adults performed a transitive reasoning task, subsequent to undergoing anatomical magnetic resonance imaging (MRI) brain scans. Behaviorally, adults reasoned more accurately than did the young adolescents. Neural results showed (i) less grey matter density in superior parietal cortex in the adults than in the young adolescents, possibly due to a developmental period of synaptic pruning; (ii) improved performance in the reasoning task was negatively correlated with grey matter density in superior parietal cortex in the adolescents, but not in the adult group; and (iii) the latter results were driven by the more difficult trials, requiring greater spatial manipulation. Taken together, the results support the idea that during development, regions in superior parietal cortex are fine-tuned, to support more robust spatial manipulation, resulting in greater accuracy and efficiency in transitive reasoning.

## Introduction

Relational reasoning is the ability to consider and manipulate relationships between multiple mental representations. One important manifestation of relational reasoning is transitive inference, that is, the process of examining and comparing a number of relational pairs in order to understand overall group hierarchy (e.g., Ralph is braver than Tim, Celia is braver than Ralph; therefore Celia is braver than Tim). This capability has been shown in human children as young as 4 years old (Bryant & Trabasso, 1971) and (arguably) is even available to many animal species, such as monkeys, rats and pigeons (Delius & Siemann, 1998). See Bermudez (2002) and Goel (2015) for dissenting opinions.

Cognitive theories of reasoning suggest that transitive inference largely relies upon spatial representations of the world (Goodwin & Johnson-Laird, 2005; Mani & Johnson-Laird, 1982). For the above example, such a spatial representation would be of the form “Celia–Ralph–Tim.” The mental scanning of this spatial model would allow one to determine whether Celia is braver than Tim.

Consistent with these cognitive theories, neuropsychological data show that the posterior parietal cortex, a region widely implicated in visual–spatial abilities (Amorapanth, Widick, & Chatterjee, 2010; Husain & Nachev, 2007; Sack, 2009), plays a key role in transitive inference. Several brain imaging studies report involvement of parietal cortex in transitive inference (Goel & Dolan, 2001; Goel, Makale, & Grafman, 2004; Wendelken, 2015; Wendelken & Bunge, 2010). A study involving neurological patients showed that patients with focal lesions to parietal cortex (BA 7, 40) have greater impairment in transitive reasoning than patients with focal lesions to prefrontal cortex (Waechter, Goel, Raymont, Kruger, & Grafman, 2013). These studies point to a critical role of parietal cortex in linguistically presented transitive inference arguments. Qualitative (Goel, 2007) and quantitative (Prado, Chadha, & Booth, 2011) reviews of the literature confirm the involvement of parietal cortex in linguistically presented transitive reasoning arguments across a large number of studies (see discussion section for qualification of these claims).

The issue of development of transitive inference remains insufficiently studied. In the behavioral literature, the seminal studies by Piaget (1921) have been followed by several works investigating developmental changes in transitive reasoning. For example, Sternberg (1980) found that response latencies and error rates in three-term transitive tasks decreased across age groups from 8 to 16 years. In a similar manner, Kallio (1982) found an ontogenetic increase of correct responses in five-term transitive inference tasks, using groups of children and adolescents from 4 to 18 years. Similar results have been obtained when comparing young adults with children ranging from 8 to 22 years (Mims, Cantor, & Riley, 1983), and also when focusing on narrower age ranges: 4–6 years (Andrews & Halford, 1998), 3–8 years (Andrews & Halford, 2002) and 6–9 years (Markovits & Dumas, 1999). Taken together, these studies suggest a gradual development of transitive inference ability throughout childhood and adolescence to adulthood.

The few developmental neuroimaging studies (Crone et al., 2009; Dumontheil, Houlton, Christoff, & Blakemore, 2010; Ferrer, O'Hare, & Bunge, 2009) have led to the proposal that the versatile reasoning skills observed in humans can be traced back to developmental (and also evolutionary) changes in a lateral frontoparietal network (Vendetti & Bunge, 2014). It should be noted that most of these studies target other kinds of relational reasoning (e.g., Raven’s Progressive Matrices or analogy tasks), not transitive reasoning per se.

Given the above literature, one might predict that maturation of parietal cortex may be related to development of transitive inference. To test this hypothesis, we undertook a Voxel-Based Morphometry (VBM) study (Ashburner & Friston, 2000) to track developmental changes in grey matter (GM) in parietal cortex, and its relation to performance in transitive reasoning tasks in adolescent and adult populations. Based upon previous research on transitive reasoning and development (Andrews & Halford, 1998, 2002; Kallio, 1982; Sternberg, 1980), we expected to find improved performance in transitive inference in the adult group. Given the consistent activation of parietal cortex reported in a number of imaging studies on transitive reasoning (see above), we expected that the behavioral changes would be associated with neuroanatomical changes in the inferior and the superior parietal cortex.

## Materials and methods

### Participants

Two groups of participants, recruited from high school and university students, took part in the experiment. The first group consisted of young adolescents with an age range of 11 years and 2 months to 16 years (*N* = 35, 18 male, 17 female). They completed the Self-Rating Scale for Pubertal Development (Petersen, Crockett, Richards, & Boxer, 1988), and age was found to be significantly associated with puberty stage (*r* = .817, *p* < .001). The second group consisted of adults with an age range of 20 years and 1 month to 24 years and 4 months (*N* = 41, 22 male, 19 female). Participant ages were normally distributed around a mean of 14 years (SD =14.1 months) for the adolescents and a mean of 22 years (SD =14.4 months) for the adults (with no statistical outliers). All participants were right-handed and did not have a history of neurological or psychiatric disorder. They all gave their written informed consent or assent. The study was approved by the local Ethics Committee (University of La Laguna) and was conducted in accordance with the Declaration of Helsinki.

An index of IQ for each participant was extrapolated from three subtests (digit span, matrix reasoning and similarities) of the Wechsler Intelligence Scale (adult version for ages > =16, children’s version for ages <16). This extrapolation was performed according to the WAIS/WISC manual guidelines (TEA, 1999; Wechsler, 2005). Due to errors in data collection, the digit span and similarities subtest scores were missed for one participant, and the similarities subtest scores were missed for two other participants. Because the IQ index for each participant was necessary for further magnetic resonance imaging (MRI) analyses (explained below), the four missing subtest scores were imputed using the expectation maximisation algorithm (Dempster, Laird, & Rubin, 1977) in SPSS 19.0.0, and then the corresponding indexes of IQ were calculated (see below).

### Task

#### Stimuli

Twenty three-term relational arguments, controlling for the use of spatial and non-spatial relational terms, and level of argument difficulty, were generated. These manipulations are discussed below and illustrated in examples A–D. The full list of arguments appears in Appendix A. Such arguments have been used in several previous imaging and patient studies of transitive inference (Goel & Dolan, 2001; Goel et al., 2004, 2007; Goel, Stollstorff, Nakic, Knutson, & Grafman, 2009; Stollstorff, Vartanian, & Goel, 2012; Vartanian, Goel, Tierney, Huey, & Grafman, 2009; Waechter et al., 2013).

The transitive terms involved either explicit spatial relations such as “inside” or “on top of,” as in examples A and B, or non-spatial relations such as “more energetic” and “louder than,” as in examples C and D.

A)

The teacher is in front of the desk;

The desk is in front of the chair;

The teacher is in front of the chair.

B)

The plates are on top of the napkins;

The cups are on top of the plates;

The napkins are under the cups.

C)

Dogs are more energetic than horses;

Hamsters are more energetic than dogs;

Hamsters are more energetic than horses.

D)

Swallows are louder than king bats;

Swallows are quieter than blue herons;

Blue herons are louder than king bats.

Argument difficulty was manipulated by either maintaining or inverting (or negating) transitive relations. For example, arguments A and C consistently use the same relations (respectively, “front of” and “more energetic”), throughout the arguments. In argument B, the relation “on top” is switched to the inverted form “under” in the conclusion, while in argument D, the relation “louder” is switched to the inverse relation “quieter” in the second premise. The switching of relations makes the task harder by making it more difficult to construct a mental model or representation of the argument, by requiring either a model containing different relations or inverting propositions. For example, the conclusion in B might be mentally turned around to read “the cups are on top of the napkins,” while in D, the second premise might be inverted to read “blue herons are louder than swallows.” Similarily, the negation of a relation (e.g., “is” vs. “is not”) also requires additional steps in constructing and monitoring mental representations. Such mental manipulations require greater cognitive resources and make these trials more difficult (Waechter et al., 2013; Waltz et al., 1999).

**Figure 1.**
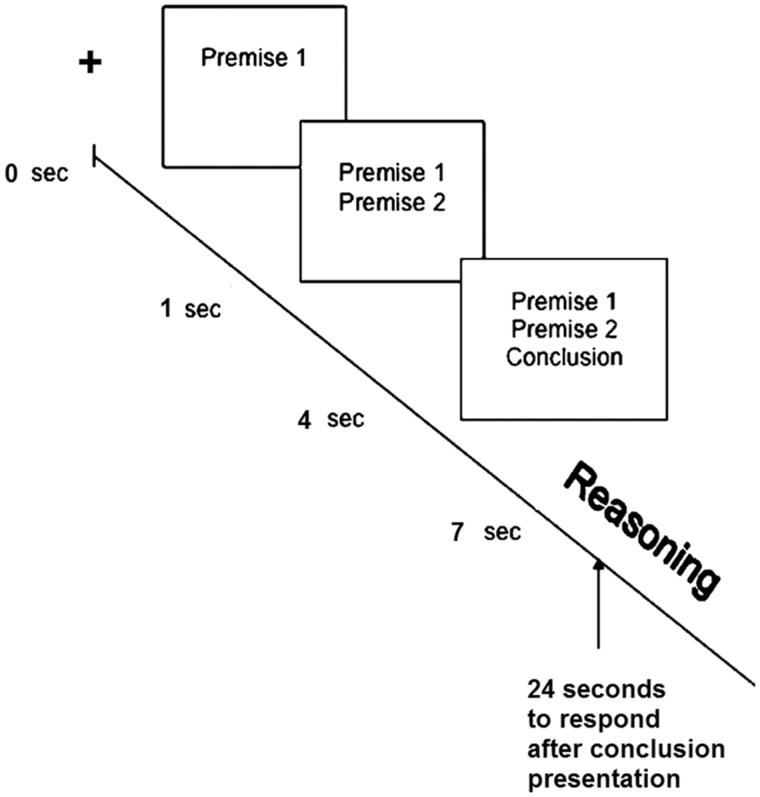
Time course of stimuli presentation.

Arguments were presented randomly on a computer screen. The beginning of every trial was signaled by a fixation cross in the middle of the screen (Figure 1). The sentences appeared on the screen with the first sentence appearing at 1 s, the second at 4 s, and the last sentence at 7 s. All sentences remained on the screen until the end of the trial. Subjects had 24 s after the presentation of the third sentence to respond. The response button triggered the next trial.

Subjects were given an explanation of logical validity along with several examples. Once they understood the concept of validity, they were given the task and instructed (in writing) as follows: “Now you will begin the experiment. If you have any questions, please ask the experimenter. Remember that you have to read carefully and try to respond as best you can. Your task is to determine if the conclusion is valid or invalid, that is, if it follows from the two premises or not.” Subjects responded “yes” or “no” by pressing a key on a computer keypad after the appearance of the last sentence. Key order was counterbalanced: half of the participants YES/NO, half NO/YES. Their accuracy scores (see below) make it clear that subjects understood the task instructions.

### MRI acquisition and analysis

High resolution sagittally oriented whole brain T1-weighted images were collected using a 3 Tesla Signa HD MR scanner (GE Healthcare, Waukesha, WI). A 3D fast spoiled-gradient-recalled pulse sequence was acquired (TR =8.7 ms, TE =1.7 ms, flip angle =12°, matrix size =250 × 250 pixels, 0.976 × 0.976 mm in plane resolution, spacing between slices =1 mm, slice thickness =1 mm). Images were visually inspected for quality control and reacquired if necessary (e.g., presence of motion artifacts).

We aimed for all participants under the age of 21 to be scanned within 90 d of their behavioral testing session. However, four participants in this age range were unable to keep this planned schedule. Therefore, the average delay between participants’ behavioral testing and scanning sessions for those participants under 21 years was 58 d (median = 54, range = 8–178). Excluding those four participants mentioned above, the delay averaged 48 d (median = 51, range = 8–87). Across all participants (i.e., aged 11–24 years) the average was 78 d (median = 64, range = 5–179). The correlation between age at behavioral session and age at scanning session was *r* = .999, which indicated that the relative age distances between participants were preserved across behavioral testing and scanning sessions. Therefore, we chose to use the age at the time of the scanning session for both the behavioral and the MRI analyses.

The structural MRIs were preprocessed and analysed with Statistical Parametric Mapping software in Matlab2013b (SPM12; Wellcome Trust Centre for Neuroimaging at UCL, London, UK). The images were segmented into six different tissue classes (grey, white, cerebrospinal fluid, bone, soft tissue and air/background) using a two-phase method. Initial segmentation was performed using SPM12’s default averaged-sized MNI template (TPM), with “very light” bias regularisation (0.0001), 40 mm cutoff for bias FWHM, and a Markov Random Field cleanup with a strength of 0.15. After this initial segmentation, a custom template was made using Template-O-Matic (TOM8, matched-pairs approach; Wilke, Holland, Altaye, & Gaser, 2008), which allowed us to create a new template based on the average age of our own participants (thereby reducing registration bias associated with using an adult reference). Segmentation of the raw images was then performed again, with the same parameters as used in the initial segmentation, but using the custom template. Raw and segmented images were visually inspected, and it was confirmed that there were no obvious artifacts or mis-segmentations. Custom-segmented grey matter images were then registered to each other and normalised to MNI space using Dartel non-linear registration with voxel sizes of 1.5 mm^3^ and preserving grey matter concentration. These spatially normalised grey matter images were then smoothed with a Gaussian kernel of 5 mm full width at half maximum and then taken forward to a SPM group analysis.

**Figure 2.**
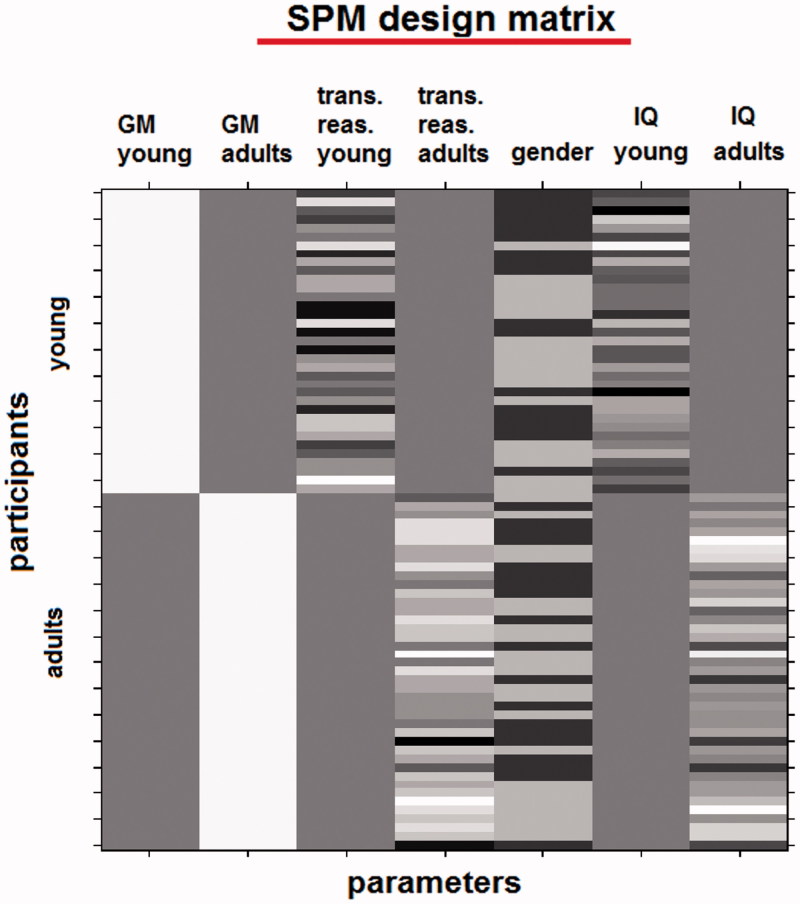
The SPM design matrix used in this study consists of seven parameters (columns): two for the grey matter images of the adolescents and the adults, two for the transitive reasoning scores of the adolescents and the adults, and three parameters of no interest (to remove the influence of gender, adults IQ, and adolescents IQ). This model allows testing for different regression slopes in adolescents and adults between grey matter density and transitive reasoning scores. Every row corresponds to the data of one participant. In the IQ and reasoning columns (i.e. columns 3, 4, 6 and 7), darker cell colors depict lower scores (brighter cell colors depict higher scores). In the gender column, dark grey cells depict women and light grey cells depict men. (Note: The first and second columns depict the grey matter images and no color code is applicable there. The big dark gray rectangles depict values that are not applicable to be modeled because they correspond to the other age group).

Statistical analyses were performed using a full factorial design investigating the interaction between the factor Age Group and the covariate transitive reasoning. This type of analysis (interaction model) tests for different regression slopes in adolescents and adults between GM density and transitive reasoning accuracy. Because we expected variance to differ between samples, we applied a non-sphericity correction. Gender and IQ were included as regressors of no interest (Figure 2) for reasons noted below. Given that our main focus is the interaction between group and reasoning accuracy, we incorporated two regressors coding for the interaction between IQ and group to ensure that the results are not driven by differences in the relationship between IQ and grey matter density between groups. Because unmodulated VBM analysis was used (i.e., preserving GM concentration), total intracranial volume was not included as a regressor of no interest, due to the inherent correction for brain volume provided by spatial normalisation.

**Figure 3.**
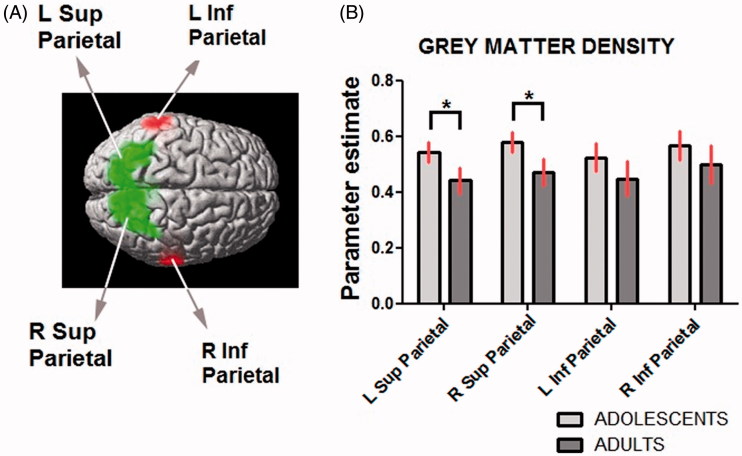
(A) The four parietal regions of interest used in this study. Two regions were placed in the superior parietal cortex (left BA7; right BA7) and two regions placed in the inferior parietal cortex (left BA40 and right BA40). (B) Parameter estimates extracted from superior parietal ROIs show significantly lower grey matter density in the adult group compared to the adolescent group (GM young > GM adults *t*-test (see also Table 2); * *p <* .05, Bonferroni corrected for multiple comparisons). Error bars depict 90% confidence intervals.

After specifying the SPM model, we used the MarsBar toolbox (Brett, Anton, Valabregue, & Poline, 2002) for region of interest (ROI) analysis. This way, differences in grey matter density between age groups, and interactions between age group and reasoning, were tested in four anatomical ROIs. Two regions were placed in the superior parietal cortex (left BA7 and right BA7) and two regions were placed in the inferior parietal cortex, comprising the supramarginal gyri (left BA40 and right BA40; see Figure 3). As noted above, these parietal regions were chosen for their critical role in transitive inference, as indicated by neuroimaging and patient studies on linguistically-presented three term transitive arguments (Goel, 2007; Goel & Dolan, 2001; Goel et al., 2004; Knauff, Fangmeier, Ruff, & Johnson-Laird, 2003; Prado et al., 2011; Prado, Van Der Henst, & Noveck, 2010; Waechter et al., 2013).The anatomical ROIs were created using the IBASPM 71 Atlas in WFU PickAtlas (Maldjian, Laurienti, Kraft, & Burdette, 2003).

## Results

### Behavioral results

First we tested for possible differences in the composite IQ index (as described in Methods) using a 2 (Age Group: adults, adolescents) × 2 (Gender: male, female) between-subjects design ANOVA. There was a significant main effect of Age (F_1, 69_ = 10.28, *p* = .002, η_p_^2^ = .130), with adults having significantly higher IQ (mean 101.38, SE 2.25) than adolescents (mean 89.79, SE 2.84). There was also a significant main effect of gender (F_1, 69_ = 6.52, *p =* .013, η_p_^2^ = .086), with males having higher IQ (mean 100.59, SE 2.37) than females (mean 91.01, SE 2.82). There was no significant Age Group × Gender interaction (*p* = .188). We therefore used IQ and gender as covariates in subsequent analysis.

A repeated measures ANCOVA with task difficulty (inverted, not inverted) and relation type (non-spatial, spatial) as within-subjects factors, age (adolescents, adults) as a between-subjects factor, and IQ and gender as covariates, revealed a main effect of age on accuracy scores (F_1, 69_ = 4.99, *p* = .029, η_p_^2^ = .067), with adults performing more accurately than adolescents, and also a trend-level main effect of Task Difficulty on accuracy scores (F_1, 69_ = 3.01, *p* = .087, η_p_^2^ = .042), with the easier trials having higher accuracy (mean proportion correct .86, SE .13) than the difficult items (mean proportion correct .78, SE .15). There was no significant main effect of relation type (*p* = .781), and all interactions were non-significant. All accuracy scores are reported in Table 1.

**Table 1.** Accuracy scores on the transitive reasoning task, considering task difficulty (inverted/not inverted items) and relation type (spatial/non-spatial items).

Type of items	Group	Mean	SD	*N*
Non-spatial inverted	Adolescents	.70	.20	33
	Adults	.84	.18	40
	Total	.78	.20	73
Non-spatial not inverted	Adolescents	.86	.18	33
	Adults	.92	.13	40
	Total	.89	.16	73
Spatial inverted	Adolescents	.73	.22	33
	Adults	.82	.16	40
	Total	.78	.19	73
Spatial not inverted	Adolescents	.76	.19	33
	Adults	.86	.17	40
	Total	.81	.19	73

### MRI results

Analyses were restricted to ROI in the parietal cortex (Figure 3, and Table 2), identified as noted above.

First, we tested for GM density differences in these ROIs, and found that the adult group displayed significantly lower grey matter density compared to the adolescent group in the left and right superior parietal regions.

**Figure 4.**
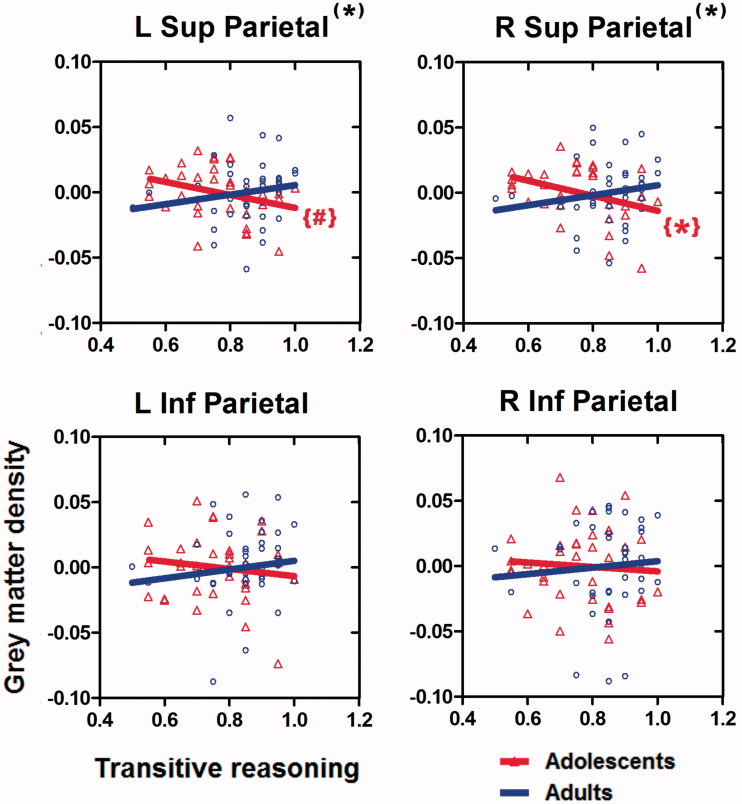
The region of interest analysis showed a significant interaction in the left and the right superior parietal regions (*), with significantly different regression slopes (of the regression lines between GM density and transitive reasoning accuracy) for the adults and the adolescents. There was no significant interaction in the inferior parietal regions. Red color is used to indicate the regression lines for young adolescents, whereas blue color is used to indicate the regression lines for adults. A significantly negative regression slope for the adolescents in the right superior parietal region was found {*}; the same contrast for the left superior parietal region was at a trend level {#}. Triangles (for adolescents) and circles (for the adults) show the adjusted grey matter density values. *p < .05, #*p <* .10; Bonferroni corrected for multiple comparisons.

Second, the relationships between reasoning performance and grey matter density were examined. ROI analysis (Table 2) showed a significant interaction effect in both left and right superior parietal ROIs with significantly different regression slopes (of the regression lines between GM density and transitive reasoning accuracy) for the adults and the adolescents. The results for the inferior parietal ROIs were not significant. These results are plotted in Figure 4.

**Table 2.** Region of interest analysis (contrasts and *p*-values; *p < .05, ^#^ *p* < .10, Bonferroni corrected for multiple comparisons).

Contrasts \ ROI	Superior parietal L	Superior parietal R	Inferior parietal L	Inferior parietal R
GM young > GM adults	.012*	.004*	.276	.537
Interaction: age group × reasoning (adults > young adolescents)	.048*	.028*	.500	.904
regression slopes (GM / reasoning)				
adolescents <0	.072^#^	.040*	.488	.692
adults >0	.144	.130	.360	.520

The reverse contrasts (e.g., adults <0) revealed no significant differences in any of the four ROIs.

Follow-up analysis of the relationships between reasoning performance and grey matter density within each group were carried out (Table 2). A significant negative regression slope was found in the right superior parietal ROI for the adolescent group (the same contrast for the homologue left superior parietal displayed a trend in the same direction). The slopes of the regression lines within the adult group were not significantly different from zero.

Third, because the behavioral analysis showed a trend-level main effect of task difficulty on accuracy scores, with higher accuracy for the non-inverted (easy) items, and also because this is a well-known effect reported in the literature (Waechter et al., 2013; Waltz et al., 1999), a second SPM model was created, taking into account task difficulty on reasoning accuracy. This model was similar to the one explained above, but here instead of using a single covariate for the transitive reasoning scores, two separate covariates were included, one for the scores on the inverted (difficult) items, and the other for the scores on the non-inverted (easy) items.

For the inverted (difficult) items, ROI analysis (Bonferroni corrected for multiple comparisons) showed a significant interaction effect in both left (*p* = .020) and right (*p* = .027) superior parietal regions, with significantly different regression slopes for the adults and the adolescents. A significant negative regression slope (*p* = .038) was found for the adolescents in the left superior parietal ROI (*p* = .054 for the right superior parietal). No significant results were found in the reverse comparisons, nor in the inferior parietal ROIs. All these tests were repeated for the non-inverted (easy) items, but no significant results were obtained. These results suggest that the results reported in the first SPM model (i.e., the relations between GM and overall transitive scores) were driven by the inverted (difficult) items.

Finally, we carried out an exploratory whole brain analysis, at the request of a referee. No significant corrected results were found (FDR, *p =* .05, minimum cluster size [k] = 5). Uncorrected results, thresholded at *p =* .001, k = 5, are reported in Supplementary material Figure S1.

## Discussion

In this study, we utilised voxel-based morphometry to investigate neural correlates of developmental changes in transitive reasoning in adolescent and adult populations. Such developmental neuroimaging studies contribute to our understanding of developmental changes in brain–behavior relationships.

At the behavioral level, we found that participants understood the task and performed at levels consistent with those obtained by related reasoning studies using the same type of task and materials (Goel & Dolan, 2001; Goel et al., 2007, 2009; Waechter et al., 2013). Adults performed better in the transitive inference task than did the young adolescents. This is an expected result, consistent with previous studies based upon transitive (Mims et al., 1983) and other relational reasoning tasks, e.g., the Raven’s progressive matrices task (Crone et al., 2009). This result is also consistent with literature that has suggested a gradual development of transitive inference ability throughout childhood and adolescence to adulthood (Andrews & Halford, 1998, 2002; Kallio, 1982; Markovits & Dumas, 1999; Sternberg, 1980).

Both adults and adolescents found trials with “non-inverted” relational terms easier than trials with inverted relations. Again, this is an expected result (Waechter et al., 2013; Waltz et al., 1999). There was, however, no effect of spatial relational terms versus non-spatial relational terms. This may be due to the fact that the non-spatial terms are easily mapped onto spatial counterparts. For example, “prettier than” or “harder than” imply “more than” and are naturally mapped onto linear spatial scale terms, while terms such as “thoughtful” and “sensitive” are accompanied by qualifier terms such as “more” or” less” which again facilitate mapping onto a linear spatial scale.

The behavioral differences between adults and adolescents were accompanied by corresponding differences in structural brain measures of GM density. Changes in structural measures are consistent with the finding that the human brain undergoes a very prolonged maturation process, with different regions undergoing substantial structural changes, at different time periods (Stiles & Jernigan, 2010). Some of these changes are related to variations in synaptic density, that is, the number of synapses per unit volume of brain tissue. Histological studies have shown that synaptic density increases after birth (due to a process of synaptic proliferation called synaptogenesis), and is followed by a period of synaptic elimination or pruning, where the most frequently used connections are strengthened and infrequently used connections are removed; this process is believed to enhance the efficiency of brain circuits (Blakemore & Choudhury, 2006). These histological findings have been replicated by structural neuroimaging studies on GM density (Giedd, 2008; Lenroot & Giedd, 2006), showing a general pattern in which GM reaches a peak during childhood and subsequently declines during adolescence and adulthood. (It should be noted that GM density in MRI is an indirect measure of a complex structure that includes not only neurons with dendritic and synaptic processes, but also glia and vasculature; thus GM loss can be affected by other maturational processes in addition to the synaptic pruning [Gogtay et al., 2004]). The developmental trajectories of grey matter are region specific, and in the parietal cortex appear to follow an inverted U-shaped curve which peaks in late childhood (at age 7.5 years in girls and 9 years in boys; Giedd, 2008). As the mean ages of our groups were 14 years for adolescents and 22 years for adults, it seems reasonable to suggest that our GM density results in parietal regions reflect pruning and other developmental processes.

Furthermore, the ROI analysis revealed that a decrease in grey matter density in the superior parietal cortex was associated with an improvement in transitive reasoning performance. This claim is supported by two results, one between the two age groups and the other within the adolescents group. In terms of the former, adults reasoned more accurately than adolescents and had less GM density in parietal lobes than adolescents (Table 2, Figure 3). In terms of the latter, more accurate reasoning in the adolescents group was related to decreased GM density (Table 2, Figure 4). In both cases, this relationship was negative, consistent with the idea that pruning is a critical feature of brain maturation, benefiting the reasoning processes.

Interestingly, there was no significant relationship between grey matter density and reasoning performance in the adult group. This may be explained by the fact that in the adolescent group, which ranged in age from 11 to 16 years, the structural developmental changes were ongoing, and levels of maturation varied with age, while in the adult group, the structural developmental changes would have stabilised. This would result in a plateauing/ceiling effect in the relationship between brain development and cognitive abilities, and there may not be enough variability for detection, given our methodology and sample size.

Given these results, the question arises: What contributions do the parietal lobes make to transitive inference? Two decades of neuroscience research on reasoning has revealed that there is no "reasoning module" in the brain (Goel, 2007). Logical reasoning is underwritten by different basic cognitive capacities, such as working and relational memory, and attention (Moses, Villate, Binns, Davidson, & Ryan, 2008; Ryan, Moses, & Villate, 2009), and is correlated with IQ measures (Stanovich & West, 2000), and modulated by belief bias, logical form, and emotions (Goel, 2007; Goel & Dolan, 2003; Goel, Navarrete, Noveck, & Prado, 2016; Prado et al., 2011). While we have tried to control for these factors through selection of IQ measures and stimuli, we cannot preclude the possibility that maturation of some non-specific cognitive abilities in parietal cortex is driving the effect.

However, our final result, that the brain–behavior relationships are driven by the (more difficult) inverted relation arguments, suggests a more specific contribution of parietal lobes to logical reasoning. As discussed in the methods section, arguments containing inverted (or negated) transitive relations require extra spatial transformations of propositions to construct and maintain mental representations/models. It is, therefore, plausible that the results obtained in these parietal regions are driven by maturation of spatial transformation abilities. This conclusion is consistent with previous findings on the involvement of visuospatial abilities in supporting relational reasoning in both human and animals (Brunamonti, Genovesio, Carbe, & Ferraina, 2011; Gazes, Lazareva, Bergene, & Hampton, 2014), and the involvement of the parietal cortex in supporting this cognitive ability (Acuna, Eliassen, Donoghue, & Sanes, 2002; Hinton, Dymond, von Hecker, & Evans, 2010).

To date, very few neuroimaging studies have focused on the relationship between reasoning and development; the few available anatomical results come from a study that investigated the neural basis of relational reasoning in children and adolescents combining fMRI and VBM techniques, utilising problems similar to the two-relations Ravens Progressive Matrices (Dumontheil et al., 2010). Unlike our results, they found developmental differences related to reasoning in the frontal rather than parietal cortex. This is an interesting difference that speaks to the tight coupling between experimental tasks and results, and the dangers of overgeneralisation.

If we construe transitive inference more broadly than it is construed here and in the cognitive reasoning literature, we find that there are actually two different sets of results in the cognitive neuroscience literature, having to do with “relational reasoning”. Studies such as ours, that have utilised linguistically-presented three-term to five-term arguments have consistently implicated parietal cortex (Goel, 2007; Goel & Dolan, 2001; Goel et al., 2004; Knauff et al., 2003; Prado et al., 2010, 2011; Waechter et al., 2013). Studies that have utilised pictorial counterparts of these arguments (Fangmeier, Knauff, Ruff, & Sloutsky, 2006; Wendelken & Bunge, 2010) or pictorially presented relational problems similar to the Raven’s Progressive Matrices problems set (Dumontheil et al., 2010), have implicated rostral lateral prefrontal cortex (BA 10).

This issue has been discussed at some length by Waechter et al. (2013). After analysing the different tasks, they conclude that the use of linguistic stimuli requires greater effort and resources to map the stimuli onto spatial mental models/representations as a prerequisite to solution (Johnson-Laird, 1986; Mani & Johnson-Laird, 1982). This places greater demands on parietal cortex (Cohen et al., 1996; Goel & Dolan, 2001; Knauff et al., 2003; Zacks, 2008). In the case of the pictorial stimuli, this mapping has already been done in the task presentation, rendering the involvement of parietal cortex less critical and perhaps shifting processing to the prefrontal cortex.

In terms of parietal cortex involvement, we hypothesised a relationship between GM in both inferior and superior parietal and transitive reasoning performance. However, we found significant results only in superior but not in inferior parietal regions of interest. There are several possible explanations for this divergence. One possibility is that the developmental changes that happen in the inferior parietal cortex have a different temporal trajectory than those that happen in the superior parietal cortex, as indicated by previous structural MRI studies (Lenroot & Giedd, 2006; Shaw et al., 2008).

A second possibility is that the roles of these areas are slightly different. Specifically, transitive inference largely relies upon visuospatial representations of the world (Goodwin & Johnson-Laird, 2005; Mani & Johnson-Laird, 1982) and superior and inferior parietal areas may be playing distinct roles in creating the visuospatial representations that subserve such inferences. In this sense, our results are consistent with a recent meta-analysis study (Wendelken, 2015) that has demonstrated a notable preferential engagement of superior parietal lobes during visuospatial processing and spatial attention tasks, compared to inferior parietal lobes. These findings are also consistent with the results of the developmental functional studies on reasoning mentioned above: when the task requires more spatial processing, activation differences between the older and the younger participants tend to appear in the superior parietal lobe (Eslinger et al., 2009), but when the spatial processing is not as important for the task, these activation differences tend to appear in the inferior parietal (Wendelken, O'Hare, Whitaker, Ferrer, & Bunge, 2011), or just outside the parietal lobe (Dumontheil et al., 2010).

Third, the null result in inferior parietal cortex may simply be due to a lack of statistical power that perhaps could be overcome by using a larger sample size. These three proposed explanations for the different results obtained in the inferior and the superior parietal regions are not mutually exclusive, and further imaging research, including developmental functional studies focused on transitive reasoning, will help to shed light on this issue.

## Conclusion

Our study provides support for a relationship between developmental grey matter changes in superior parietal cortex and transitive reasoning performance. In the context of other accompanying literature, particularly lesion data showing that focal parietal lesions result in impaired performance in transitive reasoning, we tentatively conclude that developmental structural brain changes in superior parietal cortex lead to improved performance in transitive reasoning, perhaps through the maturation of spatial transformation abilities.

## Appendix Appendix A. Transitive inference items presented in the study.

**Table:**

Premise 1	Premise 2	Conclusion
Sediment is grittier than lava	Nitro rock is not as gritty as lava	Nitro rock is grittier than sediment
Mary is a better orthodontist than Jill	Mary is not as good an orthodontist as Scott	Scott is not as good an orthodontist as Jill
The cup is darker than the saucer	The table is darker than the cup	The saucer is darker than the table
The gymnast is on the box	The box is on the ground	The gymnast is under the ground
Grapes are outside of the basket	Figs are inside the basket	Figs are with the grapes
Bert is more thoughtful than Tom	Bert is more thoughtful than Sal	Tom is not as thoughtful as Sal
Oranges are lighter than the baskets	Pears are lighter than the baskets	Pears are lighter than the oranges
The elastic is on the book	The bag is under the elastic	The book is under the bag
The thief is inside the house	The bedroom is inside the house	The thief is inside the bedroom
The athlete is behind the barbell	The dumbbell is behind the barbell	The athlete is behind the dumbbell
Swallows are louder than king bats	Swallows are quieter than blue herons	Blue herons are louder than king bats
The AI lab is more crowded than the Roth Centre	Cedar Hall is less crowded than the Roth Centre	Cedar Hall is less crowded than the AI lab
Adolescents are more sensitive than children	Adults are more sensitive than adolescents	Adults are more sensitive than children
Dogs are more energetic than horses	Hamsters are more energetic than dogs	Hamsters are more energetic than horses
Jill is prettier than Heather	Sarah is prettier than Jill	Sarah is prettier than Heather
The dishes are on top of the pans	The pots are on top of the dishes	The pans are under the pots
The plates are on top of the napkins	The cups are on top of the plates	The napkins are under the cups
The book is on the shelf	The book is under the pen	The pen is above the shelf
The teacher is in front of the desk	The desk is in front of the chair	The teacher is in front of the chair
The stapler is inside the drawer	The staples are inside the stapler	The staples are inside the drawer
